# Recruiting the “Heavy-Using Loyalists of Tomorrow”: An Analysis of the Aims, Effects and Mechanisms of Alcohol Advertising, Based on Advertising Industry Evaluations

**DOI:** 10.3390/ijerph16214092

**Published:** 2019-10-24

**Authors:** Nason Maani Hessari, Adam Bertscher, Nathan Critchlow, Niamh Fitzgerald, Cécile Knai, Martine Stead, Mark Petticrew

**Affiliations:** 1 Faculty of Public Health and Policy, London School of Hygiene and Tropical Medicine, London WC1H 9SH, UK; nason.maani-hessari@lshtm.ac.uk (N.M.H.); cecile.knai@lshtm.ac.uk (C.K.); 2 Division of Health Policy and Systems, School of Public Health and Family Medicine, Health Sciences Faculty, University of Cape Town, Anzio Rd, Observatory, Cape Town 7925, South Africa; abertscher@gmail.com; 3 Faculty of Health Sciences and Sport, Institute for Social Marketing, University of Stirling, Stirling FK9 4LA, UK; nathan.critchlow@stir.ac.uk (N.C.); niamh.fitzgerald@stir.ac.uk (N.F.); martine.stead@stir.ac.uk (M.S.); 4 SPECTRUM Consortium, University of Edinburgh, Edinburgh EH8 9YL, UK

**Keywords:** advertising, marketing, alcohol, public health, commercial determinants of health

## Abstract

Restricting alcohol advertising and marketing is a cost-effective intervention for reducing alcohol harms. However, the alcohol industry maintains that advertising does not affect consumption, claiming that its purpose is to help consumers choose brands, it is not aimed at young people, it only promotes “responsible consumption”, and any relationships with consumption are not causal. We reviewed 39 case studies (1981–2016) published by the advertising industry, which evaluate the effects of alcohol advertising campaigns. We used these to examine these industry claims. 30/39 (77%) of the case studies mentioned increasing/maintaining market share as an objective, or used this to assess the effectiveness of advertising campaigns. Most (25/39, 64%) found that campaigns increased consumption-related outcomes. Some campaigns targeted women, and heavy drinkers (e.g., Stella Artois lager, Famous Grouse whisky). Campaigns often (13/39, 33%) targeted younger drinkers. These data show that advertising does influence market share. Other effects reported in the case studies include changing the consumer profile towards: younger drinkers, women, new/lapsed drinkers, and heavy drinkers. They also present evidence of a causal relationship between advertising and consumption. In conclusion, this analysis, based on industry data, presents significant new evidence on (i) the effects of alcohol advertising on consumption-related outcomes, and (ii) the mechanisms by which it achieves those effects.

## 1. Introduction

Alcohol advertising is an important influence on alcohol consumption, and the evidence supports the restriction of advertising and marketing as a cost-effective intervention for reducing alcohol harms [1]. Alcohol marketing takes place through a wide variety of venues and formats, which Jernigan et al. have noted includes a mix of activities such as traditional print and broadcast and digital media, outdoor advertising, product placements within television shows, films and song lyrics, in-store and price promotions, branded merchandise, celebrity endorsements and sporting and musical event sponsorship [2]. 

The mechanisms through which this influence is exerted have been well-documented. Alcohol marketing may result in an affective or cognitive response among consumers, through which they become receptive to marketing, and develop an interactive relationship with the specific brand. Further, perceived peer norms on drinking are related to heavy drinking and problem drinking in late adolescence and young adulthood [3,4]. It is known, for example, that marketing and advertising donot just affect consumption but also affects beliefs about the acceptability of heavy drinking. In young people, advertising aims to be engaging, so that young people like the adverts, followed by a desire to emulate the lifestyles depicted in the adverts, leading to beliefs about the positive benefits. These changes in beliefs (about the social benefits of alcohol) then are associated with changes in consumption, and the evidence so far shows that alcohol advertising does increase the likelihood that young people start drinking, and also drink more when they do start [2,4].

The effects on consumption in general are well-evidenced, though much of the evidence is associational and cross-sectional. However, a 2016 systematic review of longitudinal studies found positive associations between level of marketing exposure and level of youth alcohol consumption in twelve such studies published since 2008 [2]. The review also emphasised the importance of understanding marketing mediators, not just whether advertising works, but also *how* it works. For example, the authors note that brand recognition, including identifying oneself as a ‘brand drinker’, is a significant mediator [2]. Despite this, in many countries, government regulation of advertising and marketing is minimal. Instead, alcohol advertising is often the subject of self-regulation [4]. Such self-regulatory approaches are championed by the alcohol industry, which rejects criticisms of their self-regulatory codes, and disputes the evidence about effects on consumption, claiming that the aim of advertising is to influence market share. 

We have identified a significant new source of evidence on the effects of alcohol advertising which does not appear to have been included in previous systematic reviews. This source is a series of evaluative advertising industry case studies, which report on the effectiveness of advertising campaigns. These case studies have been published by the Institute of Practitioners in Advertising in 23 volumes from 1981 until the most recent we identified in 2016 (a 2018 volume was published subsequent to the completion of this analysis and is not included here).

This study presents the first analysis of the content and findings of these case studies. The main aims of the study were:

(i) to use these data to test five common alcohol and advertising industry claims about the purpose, and effects (or lack of effects) of alcohol advertising (see Table 1), and
(ii) to identify the main mechanisms by which alcohol advertising works, as reported in these case studies.

## 2. Materials and Methods

We first identified five common industry claims about the direct and indirect effects of advertising on consumption (Table 1, above). We identified these from industry statements or submissions to government inquiries or consultations about the impact of advertising and marketing on alcohol consumption [11,12,13,14], and industry documents [15,16,17,18]. The latter were identified from a 2009 report which analysed internal marketing documents from both producers and their advertising agencies [15,19]. These five claims are, in effect, the main hypotheses that we tested in this study.

Second, we identified a total of 39 alcohol case studies published by the Institute of Practitioners in Advertising (IPA), and the Scottish Institute of Practitioners in Advertising (thirty-one from the IPA series, and an additional eight case studies from the companion “Scottish Advertising Works” series (See Table 2)) [20,21,22]. The largest category of case study was beer, lager, stout or cider: *n* = 20 (51.3%). Spirits (e.g., whisky, gin) and similar drinks accounted for fourteen case studies (35.9%), and wine and sherry accounted for five case studies (12.8%). We identified these by obtaining all volumes of the IPA series, and selecting the alcohol case studies, excluding other products (e.g., food, and non-alcoholic beverages). The volumes were identified from the IPA website (https://ipa.co.uk/), and from the reference lists of the volumes themselves.

The case studies present mainly quantitative data on the effects of advertising campaigns on a range of sales and other outcomes. The data (often longitudinal Interrupted Time Series (ITS) data) are commonly analysed using regression models to control for other possible explanations for any effects, such as seasonality. Many of the analyses use control groups or areas (e.g., comparing regions with or without campaigns), some of the studies also present evidence on dose-response effects. Qualitative data (e.g., from focus groups) is sometimes reported. These case studies represent a key overlooked source of evidence on the primary and secondary effects of alcohol advertising, and have the potential to shed further light on the aims, effects, and mediators of alcohol advertising.

In many instances, the case studies also provide detailed descriptions of the mechanisms by which the campaigns are intended to work. These include psychological and attitudinal mechanisms (for example, by changing perceptions and beliefs about the brand, by building brand loyalty, and by influencing perceptions of the product), as well as social mechanisms (for example through influencing the drinker through their peers and friends). These case studies therefore provide an important source of industry data on the intended and actual effects of alcohol advertising, and its mechanisms.

Data extraction and analysis: We developed and piloted a data extraction template and used it to extract the following data from each case study as free text:

- Title of case study (Year, Book)
- The stated advertising objectives
- The stated effects of the campaign
- The reported methods used by the case study to show impact (e.g., regression modelling, and any qualitative data reported), and
- Any other reported effects of the campaign (e.g., changing social norms, effects in subgroups).

A codebook was developed to systematically code and categorise the data within the main themes, and to help maintain uniformity of coding during analysis. This was done by multiple authors reading and re-reading the text of all case studies and generating a list of codes relating to the main industry claims. Coding was completed initially by two authors for the first five case studies, and then each case study was coded, and data was extracted by one author, who cross-checked codes as necessary with other authors. By this means, all text relating to any consumption-related claim was identified and extracted to tables. Initially, a longer set of nine claims was identified, but to produce a focused paper, the analysis concentrates only on the five most common claims (Table 1). Examples of case study data that support or reject these claims were then identified and are discussed below. As most (96%) of the case studies related to beer/lager/stout, or spirits and similar drinks, the quoted examples are all drawn from these two categories. 

## 3. Results

A total of 41 objectives and related effects of advertising were reported in these case studies. These are summarised in the Supplementary File Table S1, with examples. A shorter summary appears in Table 3, with the effects identified from the case studies grouped into six main categories. These categories are ordered according to the level at which they occur—from individual consumer-level effects, up to market-level and societal-level effects. These categories emerged from the data, rather than reflecting a pre-existing conceptual framework.

The data from the case studies are used below to examine the five industry claims, in turn.

### 3.1. Alcohol Industry Claim #1: Advertising Primarily Affects Brand Choice/Market Share

There is consistent evidence from these case studies to support the industry claim that growing or defending market share is an important advertising objective. However, to determine whether this is the ‘primary’ effect requires consideration of its other effects (below), as it is unlikely that advertising has one effect only, and indeed there is evidence that this is not the case [4]. Overall, 30/39 (approximately 77%) of the case studies explicitly mentioned increasing or maintaining market share as an objective or use this as a measure of campaign effectiveness. Examples of this include Stella Artois:

*“Stella’s fortunes were transformed. It outperformed every other premium lager brand, with sales increasing five-fold in just seven years, its growth outpaced the market, and as a consequence its share almost doubled…whilst commanding a significant price premium over every other comparable brand”*.

[23] (Stella Artois, 1993)

*“Advertising influenced both ROS (rate of sale) and distribution…and hence its market share”* (Bud Ice, 1999).

[24]

“*Not only did we achieve our objective of maintaining share…*” (Budweiser, 2002) [25], and *“Marketing objectives: 1. Increase awareness and share…”* (Scottish Leader whisky, 2003) [26]. 

### 3.2. Alcohol Industry Claim #2: Advertising Does not, and Is not Intended to, Stimulate Consumption

If sales and volume sales can be taken as a proxy for consumption, then all of the 39 case studies present evidence that advertising affects this outcome. However, the claim was also tested using a narrower definition of consumption-related outcomes: if a case study reported that the campaign was aimed at or effective in one of the following ways, then it was taken as evidence that advertising either aimed to, or succeeded in, increasing consumption:

(1) stimulating trial of the product,
(2) increasing drinking frequency,
(3) extending the type and number of occasions on which the product can be drunk,
(4) targeting heavy drinkers,
(5) recruiting new drinkers, and/or
(6) increasing claimed usage.

Most (25/39, 64.1%) of the case studies report changes in at least one of these six outcomes. This is sometimes an explicit aim of the campaign. For example, in the case of a Magners cider campaign:

*“We did not want to just sell Magners in the warm summer months: we wanted it to be drunk throughout the year”*.

(Magners Cider, 2008) [27]

The same campaign also: *“…amplified Magner’s relaxed timeless brand values and drove winter consumption”* [27]. 

Arguably, these examples show attempts to increase market share, rather than overall consumption. Other examples are more likely to reflect increases in consumption, such as this example where Foster’s lager used a humour-based campaign. The evaluation describes how it

*“…gave permission for a ‘drinking occasion’… Blokes were lapping up the ads, but most importantly, they were lapping up Foster’s lager too…as the ads ran, sales rose, and they have continued to do so”*.

(Foster’s Lager, 2015 [28])

Campaigns which target heavy drinkers also appear to be more clearly aimed at consumption outcomes. For example, in the case of Miller Lite, which if it *“… was to be a large profitable brand we had to attract these young heavy drinkers”* [29].

### 3.3. Examples of Advertising Being Used to Stimulate Trial

One example is from the Shakers Cocktails [30] (1985) case study, whose advertising objectives were:

“To build awareness of the brand, on the assumption (based on the development research) that this would ‘naturally stimulate’ trial. Objectives were set for post-advertising brand awareness levels of 30% spontaneous and 45% prompted, and for trial levels by end of first year of 10% of all adults, 15% in 18–34 age group.”

(Shakers cocktails, 1985 [30])

An experiment reported in a Stella Artois case study also showed the effect of advertising to stimulate consumption among drinkers who did not normally like the product:

“Served blind, Stella was not significantly preferred to other draught lager brands, indeed it frequently lost to them. The reason is that Stella is one of the most bitter tasting of all lagers, and many people find a fuller, sweeter taste more to their liking. So, despite the expensive Czechoslovakian female hops, Stella is not a significantly preferred pint…until of course you put the name back on it.”

(Stella Artois, 1993 [23])

As noted above, the fact that all campaigns had increasing sales as an objective and/or main outcome is also an indirect indicator of the intended effects on consumption. There is also a possible feedback loop, so that consumer demand itself stimulates advertising. But it seems clear from the case study data that the purpose of advertising is to stimulate demand, and one common way of measuring the effectiveness of the campaigns is in terms of willingness to purchase. The Grolsch lager campaign provides one example of this:

*“The most concrete measure of increased consumer demand is rate of purchase. Grolsch’s rate has more than trebled since campaign launch…Put simply, consumers are demanding the brand more than ever”*.

(Grolsch, 2003 [31])

The use of advertising to target heavy drinkers (see below) is also indicative of the intended effect of advertising on consumption. 

### 3.4. Alcohol Industry Claim #3: Any Observed Relationship between Advertising and Consumption is not Causal

In evaluations, inferences are strengthened if longitudinal data are available, showing effects before and after the intervention, and by using data across multiple time points. They are further strengthened by the use of concurrent control or comparison groups or areas (e.g., compared to unexposed areas or to the total market, or to non-advertised competitor products) and by the control for potential confounders [32]. Such methods were used in most (23/39) of the case studies. In some case studies, experiments (from which stronger causal inferences could be drawn) show the effect of the beverage’s brand on consumption preferences (e.g., in the Stella Artois case study, which involved a blind tasting experiment, described above). 

Alternative explanations are often controlled for using econometric modelling (e.g., Guinness [33], Miller Lite [29], Murphy’s Stout [34]). Dose-response-type analyses are sometimes used (e.g., Johnnie Walker whisky [35]). Qualitative data are used to support and explore the conclusions from the quantitative data—for example, in the case of John Smith’s bitter, qualitative research was used to develop the campaign, and to understand its appeal [36].

The use of such methods is not surprising because these case studies often have the stated aim of demonstrating a causal relationship. This is sometimes made explicit, as in these examples from the Campari [37] and Archers Schnapps [38] case studies:

“This case history will show the vital role that an integrated advertising strategy, in terms of planning input, media choice and creative content, played in that growth. It will seek to demonstrate a causal relationship between advertising and Campari sales performance…”

(Campari, 1981 [37])

*“Confidence levels are over 99% that there is a causal relationship between cumulative advertising weight and penetration”*.

(Penetration = proportion of a target audience reached [39]) (Archers Schnapps, 2000 [40])

The various causal paths and mechanisms from advertising through to sales and consumption are also clearly described in many case studies. One pathway from building a relationship with drinkers through to sales is clearly articulated in the case of a Stella Artois advertising campaign:

*“Favourable attitudes have resulted in increased claimed usage…over the course of the campaign, the brand has clearly developed unparalleled relationships with drinkers, based on deeply held perceptions of quality and discernment. This has resulted in a further long-term sales effect…attempting to explain this underlying growth trend, we plotted it against every conceivable measurable factor and found that the strongest correlation was with brand affinity... We therefore suggest that brand affinity drives underlying sales growth… The model clearly shows that brand affinity is driven by brand awareness, which is in turn econometrically linked with advertising. We can therefore prove a longer-term, slow build advertising effect. According to our econometric model, this effect is worth an additional 9000 barrels, or £3.2 million revenue, per £1 million ad spend”*.

(Stella Artois, 2000 [41])

In summary, most of the case studies present detailed evidence for a causal relationship between advertising and consumption-related outcomes (e.g., sales, usage) through the use of controlled, ITS data, while controlling for other possible explanations. This does not rule out a significant effect of advertising on market share (as per Claim #1), as opposed to consumption. Claims about effects on consumption may come from other data (e.g., Claims #4 and #5 below).

### 3.5. Alcohol Industry Claim #4: Advertising Does not Promote or Condone Irresponsible or Harmful Drinking

It has been shown that the alcohol industry is dependent on harmful drinking to protect its profits, and targets heavier consumers [42,43]. There is also evidence in these case studies of advertising specifically aiming to recruit heavy drinkers, as in these three examples:

*“If Miller Lite was to be a large profitable brand we had to attract these young heavy drinkers”*.

(Miller Lite, 2004 [44])

“…Campari needed to achieve a more stable and democratic base. This meant recruiting new, younger, mass market trialists from among the socially mobile, high-spending, heavier drinkers with the discretionary income to spend drinking in pubs in the evenings.”

(Campari, 1981 [37])

*“Television was chosen as the sole medium. This was not only for reasons of cost-efficiency (although heavy drinkers do tend to be heavy ITV watchers too), but because of the nature of the advertising task”*.

(John Smith’s Bitter, 1981 [36])

However, this is most clearly shown in a Stella Artois lager case study, where the market segmentation claimed to identify three types of drinker which it was aimed at: ‘Connoisseurs’, ‘Style-seekers’ and ‘Headbangers’. For the latter group, the strength of Stella Artois is its key selling point:

*“A quality lager to a “Headbanger” is one which is stronger…The clever thing about this line (i.e., ‘Reassuringly expensive’) was that each user group could appropriate it for their own ends. The original ‘Connoisseurs’ could use it with the ingredients story to reassure themselves that the product had a distinctive flavour, the ‘Headbangers’ who knew that the duty levied on alcohol made up a large chunk of the price, could use it to reassure themselves that because it cost more it was stronger”*.

(Stella Artois, 1993 [23])

The Stella Artois advertising campaign was reported to successfully appeal to these three groups of drinkers:

*“A cluster analysis …isolated a group of late 20s/early 30s up-market drinkers with clearly affluent quality-oriented tastes (Connoisseurs), a group of younger middle-market heavy drinkers with clearly hedonistic tastes (‘Headbangers’) and a group of younger up-market drinkers, highly experimental and with fashion-oriented tastes (Style –seekers’)…the only premium brand they all consume above average is Stella Artois”*.

(Stella Artois, 1993 [23])

The need to recruit heavy drinkers appears to be particularly strong for whisky, as these consumers are their main consumer base, as shown in the Famous Grouse whisky case study. This case study highlighted the need to recruit younger drinkers who will become future heavy drinkers:

*“As is the case in many post-mature markets, whisky brands are very reliant on a small number of heavy, and increasingly ageing, consumers, to provide the majority of volume. The Famous Grouse was no exception. Our first advertising task was to protect and build this core drinker base by persuading existing consumers and drinkers of competitive blends to choose The Famous Grouse more often. In the longer term we had to attract more younger drinkers – the heavy-using loyalists of tomorrow”*.

(Famous Grouse whisky, 2006 [45])

*“The potentially disastrous implications of losing heavy drinkers had locked whisky advertising into a creative paradigm defined by past executions, the so-called ‘whisky cage’. To achieve our objectives, we needed to break out of this cage.”*.

(Famous Grouse whisky, 2006 [45])

The Scottish Leader whisky campaign (2003) also had a target market of heavy drinkers:

“Blended whisky suffers from an ageing customer profile. Figure 1 shows that 62% of heavy users are aged 55+ and 39% over retirement age. Parts of the market are literally dying off, whisky tumbler in hand….”

*“The increased volume and rate of sale, despite the diminished distribution, indicates that where the brand was available, purchase was also hugely increased – not only from existing customers buying more”. As with many other markets, the Pareto principle applies: 20% of drinkers account for 80% of sales. So, rather than struggle to make whisky appeal to younger consumers like the premium brands, we chose to focus on the core audience of heavy users. We knew that they were older. We knew they were primarily male. We knew that unlike malt users they tended to be downmarket”*.

(Scottish Leader whisky, 2003 [46])

The Archers schnapps case study describes how a new drinks category arose to exploit session drinking in women:

*“…young people in the early 1990’s turned away from “routine heavy drinking” (see p 409). Around 1995 however the % of daily drinkers went back up to 62%, including the rise of a ‘feminine session culture’, which facilitated a new drinks sector, ‘Premium Packaged Spirits’. (i.e., spirits and mixer combinations). ‘A great deal of this is amongst Archers’ core target”*.

(Archers, 2000 [40])

Other examples of this are reported in the Miller Lite (1990) [29] and Campari [37] case studies. 

### 3.6. Alcohol Industry Claim #5: Advertising has no Influence on Young People, or on Encouraging Drinking in Young People, or Underage Drinking

These case studies do not mention effects on underage drinking – it is unlikely that they would put forward data on such adverse impacts, even though it is well-documented elsewhere that advertising aims to, and is successful in, recruiting underage drinkers [15]. However the case studies frequently (13/39, 33.3%) state that ‘younger’ drinkers (and in the case of Miller Lite (1990) [29], young heavy drinkers) are an important target market. In the case of Famous Grouse whisky, as noted in the previous section, this is stated to be because these are the “*heavy-using loyalists of tomorrow*”. Many other case studies refer to the need to recruit young drinkers. There is therefore clear evidence from these case studies that advertising often targets younger drinkers, however this is not the right dataset to examine effects in *underage* drinkers.

Finally, a number of other effects were identified in the case studies (other than those which we tested against the five industry claims). These other effects include positive effects on the parent company’s share price, and organisational effects. These effects are listed in Table 3, with examples. For example, it has been claimed that advertising benefits the consumer by keeping product prices down [47]. However, there is considerable evidence from these data that the opposite is the case, with advertising helping to maintain or increase prices. For example, decreasing price sensitivity was both an explicit marketing objective and an achieved outcome of Guinness’ 2012 campaign:

*“By 2014, Guinness started to see significant drops in price elasticity in Great Britain and Ireland…The reduction in price sensitivity also helped us to stem long-term decline in volume in GB, and we saw an upturn in fortunes in Ireland”*.

(Guinness, 2016 [33])

Several case studies also describe the mechanisms by which higher prices are maintained, e.g.,

*“The premium price ideally pays for the advertising which tells the consumer the product costs more in the first place”*.

(Stella Artois, 1993) [23]

The full list of effects of advertising which are described in these case studies, and a summary of the mechanisms through which it works, are summarised in Table 3. These are categorised according to the different level of intended or achieved impact, from the individual level up to the level of the company, or above (e.g., a claimed impact at societal level). A summary of the findings relating to the five claims appears in Table 4, above.

### 3.7. Psychological and Attitudinal Mechanisms by which Advertising Works

The case studies show in particular that influencing attitudes and expectancies is a key psychological mechanism by which advertising works. Many of the case studies present qualitative data to describe how these mechanisms contribute to the success of a campaign, such as in the case of Hofmeister lager (1985): *“The in-depth interviews supported the earlier qualitative research in suggesting that Hofmeister’s new appeal lay in drinkers’ perceptions of it as a popular, fashionable lager, thus, the link between the brand’s advertising-born saliency and its sales increase was confirmed”* [48]. Advertising also helps prepare the consumer before purchase: *“That promotions have worked so well is attributable in a large part to the role that advertising plays before consumers even step into the supermarket”* (Stella Artois, 2003 [23]).

In some cases, it appears to be used to override consumers own preferences: described in the example above. *“Buyers want to feel that they are making a sensible, defensible choice that reflects well upon them as knowledgeable beer drinkers. This can override actual taste preference, the brewery adage that ‘people drink with their eyes’ has been repeatedly confirmed by blind and branded product tests, where the brand-names can reverse the preferences expressed ‘blind’”* (John Smith Bitter, 1983, [36]). 

The Stella Artois (1997, Book 9) case study provides another clear example:

*“In its Belgian homeland, Stella is a swilling lager lacking in premium credentials, in blind taste tests in the UK, the product regularly finishes bottom of the league (being too bitter for many). However, add the Stella name and the perspective of British lager drinkers changes radically… This phenomenon, where brand potency overcomes product reality, is also seen quantitatively. Stella outperforms its key competitors on almost every image dimension”*.

[49]

These data suggest that advertising both aims to change attitudes, expectancies and consumption behaviour, and is effective in doing so.

### 3.8. Lagged Effects of Advertising

There is also interesting data in these case studies on the continuing effects of campaigns even after they have finished. This is described in the case of Boddington’s (1995) beer:

*“However, the effect of advertising does not cease at this last data point. Not because we continued advertising after that period (even though we did) but because there would be residual effect from the advertising up to that period… advertising was responsible for an extra 62,618 barrels (excluding adstock) and 94,341 barrels (including adstock)”*.

(Boddington’s beer, 1995 [50])

This period can be considerable (“*the additional effect of advertising appears to continue for at least 6 years*”—Murphy’s stout (1993-5) [34]. For example, this is described in the case of Budweiser, and Johnnie Walker whisky:

“The analysis shows that, although the ads were only on air for a two and half year period, they ‘continue to generate a further 72 million bottles and £77, of extra revenue over the next six years’”

(Budweiser, 2003 [25])

*“The result has been accelerated growth that even now, eight years later, remains very strong and continues to outpace the category significantly”*.

(Johnnie Walker whisky, 2009 [35])

These lagged effects have implications for future evaluations and systematic reviews of the effects of advertising (see below).

### 3.9. Return on Investment in Advertising

Some of the case studies also report some quantitative measure of the Return on Investment (ROI) of advertising. The ROI ratios where reported range from 2–3, though in some cases are much higher (e.g., Fosters (2015): “*every £1 spent on advertising since 2010 has generated £32 in sales revenue*”) [28].

## 4. Discussion

These findings show firstly that advertising does, as the alcohol and advertising industries claim, influence share of the market. Whether this is its primary effect is difficult to ascertain, but it appears clear from these evaluations that advertising does considerably more than this, with evidence that it is used to effectively change the consumer profile, for example, towards younger drinkers, women drinkers, new or lapsed drinkers, and heavy drinkers. It appears to do this through a wide range of psychological, behavioural and social mechanisms (Table 3), by influencing drinkers directly and also indirectly through their peers and drinking environments. 

The case studies also provide consistent evidence that advertising increases both brand sales and indirect consumption measures and that this relationship is causal, with most of the studies using appropriate quasi-experimental methods and longitudinal data (including, for example, ITS methods) to support their claims of effectiveness. While the current dataset is not sufficient to draw firm conclusions on advertising’s effects on individual consumption, as distinct from shifting brand choices within a particular category, there appears to be strong evidence of its effects on consumption within specific categories of drinker. Moreover, all this is consistent with the existing evidence [51]. A recent study found that most alcohol is consumed by drinkers drinking above guideline levels, moreover, the heaviest drinking 4% of the population accounted for almost a quarter of revenue. It concluded that in England at least, the alcohol industry is highly financially dependent upon heavy drinking, across all beverage types [51]. This was disputed strongly by the alcohol industry at the time [52]. However, our findings show explicit targeting of heavy drinkers in the case of Stella Artois lagers, John Smith’s Bitter, and whisky (Scottish Leader, and Famous Grouse), as well as Archers Schnapps, with the latter targeting female ‘session drinkers’.

### 4.1. Advertising Mechanisms

These analyses of the advertising campaigns also provide insights into the causal pathways and mechanisms through which advertising works (summarised in Table 3). It is clear that advertising aims to influence a wide range of individual-level attitudinal, emotional, interpersonal and other mechanisms, and that it can also do this indirectly by affecting the wider environment – for example in the case of Magners cider, by targeting influential drinkers, and influential bars:

*“We targeted the influential drinkers who had a high degree of interest and were well connected…the on-trade teams targeted influential bars for initial distribution, and we undertook an extensive sampling campaign to accelerate trial”*.

[27]

The findings also challenge the alcohol industry argument that advertising aims to help consumers make rational informed choices, as some of the pathways bypass this, appealing directly to the emotions, even over-riding existing preferences, as shown in the blind-tasting experiment described for Stella Artois. The existence of such mechanisms is well-documented – particularly in the field of behavioural economics, and they have been applied to advertising and marketing practices [53,54]. 

Table 3, and other data on mechanisms in these case studies (such as halo effects), may provide a useful starting point for building a system-level model of alcohol advertising, which moves beyond the linear, rational choice model which the industry claims is the norm and use to dispute any causal relationship (See Box 1 for an example) [8].

Box 1 **Example of a linear, rational decision-making model of how alcohol advertising works.**

(from spirits.eu, a European spirits industry representative body)

*‘Advertising is a progress through various stages. Behaviours are only influenced by advertising at the end of a 9-step process, beginning with exposure to the ad, and moving through paying attention to it, reacting favourably, comprehending it, agreeing with it, sorting and retaining its content, remembering it, deciding upon it and behaving in accordance with this decision. At any step of this tenuous process the causal chain can be broken. Most available literature is unable to demonstrate a direct causality throughout these 9 steps.’*

The findings on the lagged effects of advertising are particularly important. They have significant implications for evaluations of marketing restrictions, as it means that null findings from any such studies can potentially be explained by the fact that the advertising clearly affects consumption for a considerable period after it is withdrawn. The implication is that the lengths of follow-up needed for evaluative studies may be much longer than anticipated, if any effect of marketing restrictions in reducing consumption are to be identified. For comparison, the lengths of follow-up in previous such evaluations ranged from 1 to 3.5 years [55]. The recent Cochrane review of the effects of alcohol advertising restrictions noted that the length of time required to establish whether a ban has been effective or not is currently unclear but noted that it can take an extended period for the effects of advertising to cease. It suggested that while there are likely to be contextual differences, it seemed reasonable to monitor the effects of any country-level ban for at least 18 months. The findings of our review suggest that much longer periods may in fact be needed. This is an important area for further research.

### 4.2. Strengths and Limitations

A significant strength of the study is that it uses a hitherto overlooked source of industry evidence that triangulates well with current scientific evidence, particularly recent evidence reviews that show the impact of alcohol advertising on consumption [1,2]. The key limitation is that these case studies are not a representative sample of advertising industry evaluations, that is, the nature of the sample means that it does not include evaluations of ineffective advertising campaigns. It is also unlikely that case studies will include examples of advertising which is clearly harmful, e.g., the targeting of underage drinkers, even though the role of alcohol marketing in youth drinking is well-documented elsewhere [56,57]. Furthermore, it is generally difficult to tell from the data whether campaigns really are competing for market share or are recruiting new drinkers – the meaning of terms like ‘new drinkers’ are unclear. A further limitation is that there is often limited methodological information in the published case studies, nor is it entirely clear what type of peer review they underwent before publication. Nonetheless, they represent an appropriate additional real-life evidence base—based on secondary data from the industry itself—for assessing the industry’s own claims about the effects of advertising, and we suggest that these case studies should be considered for incorporation into future systematic reviews, as they are mainly evaluations of fully-rolled-out national campaigns.

It should also be noted that the case studies span a long time period. One potential criticism which may be levelled at the findings is that they represent old campaigns, and do not reflect current practice. Certainly, there are few alcohol case studies dating from after the tightening of Ofcom (UK communications regulator) regulations in 2004/2005: only 2/39 case studies date from 2005 onwards. It is not possible to determine from these data alone whether subsequently revised Ofcom regulations did affect advertising, or whether they meant that advertising companies were subsequently less likely to submit alcohol case studies after 2005, and/or were less likely to report promotion of harmful/heavy drinking after this date. However, our analysis of the importance of heavy drinkers to the alcohol industry used data from 2013/14, so this dependence clearly continues [51]. Moreover, even data from older case studies is appropriate for assessing some of the key claims – for example, making a judgement about the causal relationship between advertising and consumption outcomes does not depend on the age of case studies, and the same applies to judging claims about the effects of advertising on brand share. In the latter case, there is evidence from all time periods that this is a considerable over-simplification.

## 5. Conclusions

These findings show the role that alcohol advertising has in promoting alcohol consumption and heavy drinking. This appears to extend well beyond its effects on market share—what the industry claims is its “primary” effect—and suggests that future evaluations and systematic reviews need to include evidence on the wider psychological, behavioural and social and societal mechanisms and effects of alcohol advertising – for example, by ensuring that any evaluations examine these processes and mechanisms (as well as consumption outcomes).

Finally, the claims that we examined are used by the alcohol industry to argue that tighter restriction on advertising is unnecessary because advertising does not ‘work’ to promote consumption, and, they claim, even if it did, that advertising does not promote *harmful* consumption. Our findings clearly do not support this interpretation. The study provides clear evidence, based on industry data, of the mechanisms by which advertising affects consumption, and shows its emphasis on recruiting drinkers, and particularly younger drinkers, as well as its emphasis on encouraging more drinking occasions, and, in some cases, targeting heavy drinkers. The reliance on heavy drinkers also requires a constant search for new groups of heavier consumers, to replace those that die in no small part due to that consumption; As one whiskey producer put it, “*Parts of the market are literally dying off, whisky tumbler in hand….”* These findings should be used to inform future evaluations and systematic reviews of the effects of alcohol advertising, and future assessments of the potential effects of regulatory interventions.

## Figures and Tables

**Table 1:** Five common alcohol industry claims about the effects of advertising on consumption.

Claim	Example of Claim, and Source
1. Advertising primarily affects brand choice/market share: The primary purpose of advertising is to affects brand choice, thus increasing market share (i.e., not stimulating total consumption)	*“In a mature market, the purpose of marketing is to differentiate among individual branded products—it aims to increase market share”*. [5]
2. Advertising does not, and is not intended to, stimulate consumption: Advertising does not create demand, or affect consumption	*“…advertising is very effective in achieving brand-switching but has, at most, a marginal impact on total consumption.”* *“… advertising is part of a producer response to consumer demand.”* [6,7]
3. Any observed relationship between advertising and consumption is not causal	*“There are no studies of alcohol advertising which can effectively trace the ‘effect’ of an ad from exposure through purchase to subsequent consumption behaviour. There is no reliable research which demonstrates a causal link between advertising and consumption”* [8]
4. Advertising does not promote or condone irresponsible or harmful drinking	*“The Scotch Whisky industry takes seriously its commitment to marketing products to consumers in a responsible way. Responsible advertising is fundamental for Scotch Whisky producers.… …distillers are not looking for consumers at any cost”*. [9]
5. Young people: Advertising has no influence on young people, or on encouraging drinking in young or underage people	*“Alcohol advertising and/or sports sponsorship do not target nor do they influence young people in their attitudes to drinking and drinking behaviour…”* [10]

**Table 2:** Brands evaluated in each case study, category of beverage, and year of publication. IPA: Institute of Practitioners in Advertising.

Brand/Product	Year of Publication of Case Study
IPA ‘Advertising Works’ series	
Campari: An evaluation of the effectiveness of the current Campari campaign (Vol 1)	1981
John Smith’s bitter (Vol 2)	1982
Hofmeister lager (Vol 3)	1985
Shakers cocktails (Vol 3)	1985
Paul Masson California Carafes (wine) (Vol 3)	1985
Country Manor (wine) (Vol 4)	1987
Castlemaine XXXX Lager (Vol 4)	1987
Miller Lite lager (Vol 5)	1990
Lanson Champagne (Vol 6)	1991
Croft Original sherry (Vol 6)	1991
Aberlour Whisky (Vol 6)	1991
Stella Artois lager (Vol 7)	1993
Boddingtons bitter (Vol 8)	1995
John Smith’s bitter (Vol 8)	1995
Marston’s Pedigree bitter (Vol 8)	1995
Stella Artois lager (Vol 9)	1997
Murphy’s Stout (Vol 9)	1997
Bud Ice lager (Vol 10)	1999
Bacardi Breezer (rum-based drink) (Vol 10)	1999
Famous Grouse whisky (Vol 10)	1999
Archers schnapps (Vol 11)	2000
Glenmorangie whisky (Vol 11)	2000
Stella Artois lager (Vol 12)	2003
Budweiser lager (Vol 12)	2003
Stella Artois lager (Vol 12)	2003
Famous Grouse whisky (Vol 15)	2007
Magners Cider (Vol 16)	2008
Johnnie Walker Whisky (Vol 17)	2009
Gordon’s gin (Vol 21)	2013
Foster’s lager (Vol 22)	2015
Scottish Advertising Works series (Vols 1-4)	1999-
Glenmorangie (Vol 1)	1999
Tennent Caledonian Breweries (Vol 2)	2001
Bowmore (Vol 2)	2001
Ardbeg (Vol 2)	2001
Scottish Leader whisky (Vol 3)	2003
Grolsch (Vol 3)	2003
Bowmore (earlier campaign) (Vol 3)	2003
Grolsch (Vol 4)	2005

**Table 3:** Summary of advertising’s reported effects and mechanisms of effect, from the individual level to the system level (summarised from Supplementary File, Table S1).

The Level of the Effect	Mechanisms Identified in These Case Studies	Outcomes Seen in These Case Studies Key Individual-Level Outcomes
Individual level: Psychological mechanisms	Advertising increases exposure to, and awareness of the product, through influencing the individual consumer’s attitudes, emotions, and through other psychological impacts (e.g., it can override existing preferences or beliefs). It is also described as strengthening consumer predisposition/affinity towards the brand, conveying perceptions of quality, changing attitudes, and acceptability, and “preparing” buyers before purchase.	This helps build an emotional bond with the brand, educates the consumer about the product/brand, and builds loyalty to the product or brand.
Individual level: Behavioural mechanisms	Advertising can promote changes in individual consumption behaviours: stimulate demand/consumption, stimulate trial of product, appeal to consumers friends.	Increased spend per purchaser, increased volume/frequency per customer, extension of drinkers’ drinking repertoires, increased range of drinking occasions, increased heavy use in existing drinkers (who may become heavy drinkers).
Brand/product effects	Advertising helps create/strengthen a brand and/or the product and its identity/personality/image. In particular, it can build/strengthen brand equity (brand equity: The value that a consumer attributes to a brand —e.g., reliability, quality, memorable etc.).	Through these mechanisms, advertising can reposition the product in the market, protect the brand, prevent decline in the brand, help premiumisation, support other promotions, and can be used to meet “unmet consumer need”.
Market-level effects	Advertising helps market segmentation and develops new markets, increases market share/penetration, and “share of voice”, develops new product categories, and builds the future market, increases product distribution, increases sales volume (volume growth and volume share), helps sustain growth, maintains or increases higher price/brand value, increases sales of non-advertised products, can involve spill-over to category, and can be used to block competition.	Through these mechanisms it can help recruit new drinkers, recruit younger drinkers, and increase rate of sale/purchase.
Parent company outcomes	These include the above mechanisms, advertising also creates property for future use.	Improves value of parent company, strengthens company profile, helps increase/maintain share prices.
Wider societal effects	These societal effects can be mediated through the above individual, and through market-level mechanisms.	Claimed effects in the case studies include providing employment, providing “role models”, “enhancing national pride” (e.g., “We’ve researched (Johnnie Walker’s 2008 campaign) Keep Walking all around the world, and seen it galvanise Thailand in the wake of the currency collapse, reinforce the pride in national progress of Brazil and China, provide role models for masculinity in the Middle East…”).

**Table 4:** Summary of findings regarding five key industry claims about advertising, based on advertising industry evidence.

1. Alcohol Industry claim #1: Advertising primarily affects brand choice/market share	Advertising does influence market share, it is unclear whether it is its “primary” effect as the case studies show that it has many other intended outcomes
2. Advertising does not, and is not intended to, stimulate consumption	Highly unlikely. Evidence from these case studies shows that it aims to stimulate trial, increase drinking frequency, increase range of drinking occasions, target heavy drinkers, and recruit new drinkers
3. Alcohol Industry claim #3: Any observed relationship between advertising and consumption is not causal	Unlikely. There is detailed longitudinal evidence from industry case studies that the relationship is causal
Alcohol Industry claim #4: Advertising does not promote or condone irresponsible or harmful drinking	False. There is clear evidence from some case studies that advertising successfully targets heavy drinkers
Alcohol Industry claim #5: Advertising has no influence on young people, or on encouraging drinking in young people, or underage drinking	Unclear evidence regarding underage drinking (but see main text), however, there is evidence from these case studies that advertising frequently needs to recruit, and is targeted at, younger drinkers

## Supplementary Materials

The following are available online at https://www.mdpi.com/1660-4601/16/21/4092/s1, Table S1: Range of effects of advertising identified in these case studies.
