# Supplementary material for: Recruiting the “Heavy-Using Loyalists of Tomorrow”: An Analysis of the Aims, Effects and Mechanisms of Alcohol Advertising, Based on Advertising Industry Evaluations
Source: Int J Environ Res Public Health. 2019 Oct 24;16(21):4092. doi: 10.3390/ijerph16214092 (PMC6862254; doi:10.3390/ijerph16214092)
Supplement: Supplementary file 1 [file ijerph-16-04092-s001.pdf]

## Supplementary File 1 -Appendix 1:

## Table S1. Range of effects of advertising identified in these case studies

These are arranged from individual-level up to societal level, broadly:

- Individual effects (attitudinal, emotional, behavioural)
- Effects which increase exposure to advertising
- Effects which increase availability of product
- Stimulate consumption of product in general population
- Stimulate consumption of product in heavy users
- Effects on the brand
- Effects on the wider market
- Effects on social norms (including effects on peers; providing role models)

(Full references to the 39 case studies are given in the main document)

| **Type of effect** | **Objectives and effect of advertising** | **Examples** |
|---|---|---|
| Individual-level | Increases exposure to/awareness of product | Shakers cocktails: "prompted brand awareness data showed that awareness levels corresponded with advertising (see Table 5). In the four target areas, prompted awareness was mostly >50% during the period of pre-Christmas advertising, dropping to 36-46% in the new year (no advertising)."<br><br>Country Manor wines: "Advertising objectives:<br>1. To generate a high awareness of the brand and product attributes"<br><br>Archers: "There is a consistent relationship over time between brand awareness and use'<br><br>Budweiser: "Awareness gains were clearly driven by the new advertising (Figure 22). There is a clear and immediate correlation between ad awareness and TVRs."[TV rating points]<br><br>Plus many others |
| Individual-level | Increase volume/frequency per customer | Archers Schnapps: "Our advertising strategy has succeeded primarily by growing the brand through increasing penetration, allowing us then to develop frequency among a formidable army of Archers drinkers." |

| | | |
|---|---|---|
| | | Hofmeister: “The initial advertising objective was therefore to raise Hofmeister’s profile in the market-place and to increase its franchise of frequent drinkers.<br><br>Country Manor wines: The objective set was to aim to increase frequency of purchase among existing users.<br><br>John Smiths bitter: “The 18-24s appear to be the most volatile group in terms of brand imagery and choice, and produced the greatest volume return for a given proportional change. If brand advertising could regain the loyalty of only a small proportion of these drinkers, share would regained,<br><br>Murphys: “This group was far more likely to be appreciative of an easy drinking offering; the likelihood being that these drinkers would actually increase their consumption to the benefit of Murphy’s. Non-stout drinkers also represented an enormous opportunity”.<br><br>Fosters: “Blokes were lapping up the ads, but most importantly, they were lapping up Foster’s lager too…as the adds ran, sales rose, and they have continued to do so” (p32).<br><br>Grolsch: “Not only are more households buying Grolsch, but each purchaser is now spending more money and buying more volume than they did in 2001 (purchasing 3.8 litres more and spending £8.46 more per person in 2003 than in 2001)…rate of purchase has increased from nine gallons bi-monthly in January/February 2000, to more than 18 gallons bi-monthly in 2002”.<br><br>Archers: “We can see sales growth overlapping with penetration, implying an increase in amount drunk per customer”. |
| Individual-level | Increase spend per purchaser | Grolsch: “Off-trade customers now spend more money a head on Grolsch than they do on K. That result is replicated in the on-trade…”<br><br>Bowmore: “where the brand was available, purchase was also hugely increased – not only from existing customers buying more, but also customers thwarted by the delisting seeking alternative suppliers. We believe this is strong evidence of brand equity’. |
| Individual-level | Stimulate demand/consumption | Magners: “We dropped high-impact highly visible multimedia advertising into new areas to build distribution in pubs and bars quickly. Media was deployed to generate mass consumer demand and ensure the sales teams’ distribution drive delivered maximum return”<br><br>Bacardi Breezer: “We observed strong correlations between advertising and awareness, awareness and trial, and trial and recent consumption.” |

| | | |
|---|---|---|
| | | Fosters: ‘Blokes were lapping up the ads, but most importantly, they were lapping up Foster’s lager too…as the adds ran, sales rose, and they have continued to do so”<br>Stella Artois: “Proportion claiming to drink Stella increased”<br><br>Famous Grouse: “2% increase in the number of ‘most often drinkers’ while competitors reduced by 8%.<br>“Our 55+ drinkers, disproportionately important in volume terms increased by 144,000 between 1997 and 1997” (competitors lost drinkers).” During the same period the number of 18-24 year olds “ever drinking” TFG has risen by over 10% despite a 12% drop in penetration among this age group within Scotch whisky as a whole |
| Individual-level | Increase heavy use; target heavy drinkers | Archers Schnapps: “Around 1995 however the % of daily drinkers went back up to 62%, including the rise of a ‘feminine session culture’, which facilitated a new drinks sector, ‘Premium Packaged Spirits’. (i.e. spirits and mixer combinations). ‘A great deal of this is amongst Archers’ core target’.”<br><br>Scottish Leader whisky: “As with many other markets, the Pareto principle applies: 20% of drinkers account for 80% of sales. So, rather than struggle to make whisky appeal to younger consumers like the premium brands, we chose to focus on the core audience of heavy users’. We knew that they were older. We knew they were primarily male. We knew that unlike malt users they tended to be downmarket”. And: “Blended whisky suffers from an ageing customer profile. Figure 1 shows that 62% of heavy users are aged 55+ and 39% over retirement age. Parts of the market are literally dying off, whisky tumbler in hand’”.<br><br>Miller Lite: “If Miller Lite was to be a large profitable brand we had to attract these young heavy drinkers”.<br><br>Campari: “Campari needed to achieve a more stable and democratic base. This meant recruiting new, younger, mass market trialists from among the socially mobile, high-spending, heavier drinkers with the discretionary income to spend drinking in pubs in the evenings.”<br><br>Campari: “The advertising not only transformed Campari literally into a different kind of drink, and one that appealed to a different type of person from the Campari and soda loyalist, but also succeeded in modifying people’s drinking behaviour in a dramatic way.” (p. 124)<br><br>Stella Artois: “For Stella’s advertising to work …It had to elevate Stella’s image for strength above Holsten Pils, and maintain a lead over the ‘macho’ positioned Tennents Extra. This would secure the brand’s appeal to ‘Headbangers’”.<br><br>Famous Grouse: “As is the case in many post-mature markets, whisky brands are very reliant on a small number of heavy, and increasingly ageing, consumers, to provide the majority of volume. The Famous Grouse was no exception. Our first advertising task was to protect and build this core drinker base by persuading existing |

| | | |
|---|---|---|
| | | consumers and drinkers of competitive blends to choose The Famous Grouse more often. In the longer term we had to attract more younger drinkers – the heavy-using loyalists of tomorrow.<br>The potentially disastrous implications of losing heavy drinkers had locked whisky advertising into a creative paradigm defined by past executions, the so-called 'whisky cage'. To achieve our objectives, we needed to break out of this cage." |
| Individual-level | Strengthen customer Loyalty | Boddingtons Bitter: 'The task here was to grow volume outside Granada from a small base...Penetration of Boddingtons has increased sevenfold but perhaps more significant is the increase in claimed 'most often' drinking, and increasing degree of loyalty to the brand."<br><br>John Smiths bitter: "The 18-24s appear to be the most volatile group in terms of brand imagery and choice, and produced the greatest volume return for a given proportional change. If brand advertising could regain the loyalty of only a small proportion of these drinkers, share would regained, and with that would follow benefits for total pub revenue" (Pg. 16). |
| Individual-level | Over-ride existing preferences or beliefs | Croft Original Sherry: "Despite the similarity in sweetness of Croft Original and Harveys Bristol Cream, consumers believed [as a result of the advertising] they were drinking a drier product"<br><br>John Smith: . "Buyers want to feel that they are making a sensible, defensible choice that reflects well upon them as knowledgeable beer drinkers. This can override actual taste preference; the brewery adage that 'people drink with their eyes' has been repeatedly confirmed by blind and branded product tests, where the brand-names can reverse the preferences expressed 'blind'."<br><br>Stella Artois: "In its Belgian homeland, Stella is a swilling lager lacking in premium credentials; in blind taste tests in the UK the product regularly finishes bottom of the league (being too bitter for many). However add the Stella name and the perspective of British lager drinkers changes radically…This phenomenon, where brand potency overcomes product reality, is also seen quantitatively. Stella outperforms its key competitors on almost every image dimension".<br><br>Stella Artois: 'Was the secret of Stella's success that it was stronger than other premium lagers? No. Holsten Pils was significantly stronger…Stella's mythical potency was just that – a myth!'<br><br>'Served blind, Stella was not significantly preferred to other draught lager brands, indeed it frequently lost to them. The reasons is that Stella is one of the most bitter-tasting of all lagers, and many people find a fuller, sweeter taste more to their liking. So, despite the expensive Czechoslovakian female hops, Stella is not a significantly preferred pint…until of course you put the name back on it. |

| | | |
|---|---|---|
| | | Archers: "…allowing us to continue to charging a full-spirit premium on a half-strength measure'." |
| Individual-level | Build emotional bond with brand | Fosters: "The most important ingredient in this process is emotional bonding. If people really like a brand, they're willing to pay more for it.'<br><br>Lanson: Qualitative research shows the "emotional bond built by the advertising between Lanson and many Champagne buyers".<br><br>Budweiser: "Make the brand desired. The image had to strike a chord".<br><br>Glenmorangie: "We also improved younger malt drinkers' 'feeling the brand on two key criteria: 'It's one of a number of brands which I choose' 'I'm more attracted to it than I used to be'. |
| Individual-level | Meeting unmet consumer need | Bacardi Breezer: "This is the story of how a brand with no role… created a category and transformed a company. It is about the opportunity that can flow from tapping an unmet consumer need" |
| Individual-level (psychological) | Strengthen consumer predisposition towards the brand; including perceptions of quality; attitudes, acceptability | Grolsch: 'Together these factors led to dramatic improvements in consumer predisposition towards Grolsch'<br><br>Campari: "As we were trying to modify existing attitudes and behaviour rather sharply, we wanted to use a medium capable of making an immediate impact.'<br><br>Johnnie Walker: "Such a turnaround in fortunes would require significantly stronger consumer engagement with the brand…Johnnie Walker had to change the rules of the category and play a far greater role in consumers' lives'. |
| Individual consumer-level | Stimulate trial of product | Bacardi Breezer: 'We observed strong correlations between advertising and awareness, awareness and trial, and trial and recent consumption'<br>Archers: "an adaptive advertising strategy kickstarted the brand, encouraged trial, and helped Archers weather further dramatic shifts in drinking patterns… Archers gained a total of 5,241,600 triallists (13.4% of the drinking population) during these five years, and, most importantly, a new core of 684,689 users"<br><br>Shakers cocktails: Advertising objectives: to build awareness of the brand, on the assumption (based on the development research) that this would 'naturally stimulate' trial. Objectives were set for post-advertising brand |

| | | |
|---|---|---|
| | | awareness levels of 30% spontaneous and 45% prompted, and for trial levels by end of first yr of 10% of all adults, 15% in 18-34 age group.<br><br>Miller Lite: 'In pubs…a further free pint was given away with purchase of the first, in order to encourage trial. At the same time a proportion of the cans, during launch into each area, carried a voucher entitling the holder to 60p off the next purchase of a four-pack'.<br><br>Castlemaine XXXX: Table 6 summarises the rapid growth of XXXX's standing in Yorkshire and Central on all key measures: Advertising Awareness; Brand salience; Brand Image (on product-related and user-related dimensions); Trial and drinker commitment."<br><br>Magners: "We targeted the influential drinkers who had a high degree of interest and were well connected…the on-trade teams targeted influential bars for initial distribution, and we undertook an extensive sampling campaign to accelerate trial'. |
| Individual consumer-level | Appeal to consumers friends [mechanism] | Fosters: "The image projected by the advertising must not only appeal to the individual drinker, but it must also appeal to their friends. A heavy weight of advertising is also crucial in these terms, providing reassurance of a brand's success and popularity to the young drinker"<br><br>Aberlour: 'It may well be that purchasers may want to tell their friends about the offer they have invested in and encourage them to try Aberlour" |
| Individual consumer-level | Educate the consumer about the product/brand [Surprising how little of this there is] | Shakers cocktails: 'the role for the advertising was to educate the target market by communicating the concept in a way that was empathetic to both the audience and the product' and 'to communicate the range'. |
| Individual consumer-level | Increase the range of potential drinking occasions | Paul Masson: "In addition, qualitative research conducted at regular intervals throughout the period continued to suggest not only advertising's role in the development of a highly distinctive brand personality, but also in positioning the brand as acceptable on a number of usage occasions, and against both sectors of the target audience. This too has been confirmed quantitatively" (see Table 5).<br><br>Magners: "We did not want to just sell Magners in the warm summer months: we wanted it to be drunk throughout the year so we needed a creative idea that provided a platform for a sustainable creative message that could be refreshed throughout the year'… there were also significant volume returns to be gained from the more traditionally off-peak cider periods: spring and autumn. This justified our decision to encourage Magners drinking all year round." |

| | | |
|---|---|---|
| | | Fosters: “Heineken was working on a new premium lager with an ABV of 4.8& - Foster’s Gold – to extend Fosters into new ‘upmarket’ drinking occasions”.<br>Shakers Cocktails: Test phase 2. Research with narrower group – 18-24 and 25-34 C1C2 women – to test shortlist of pack designs, brand names and flavours (no detail on sample size or method). Defined a range of potential drinking occasions for the product, e.g. parties, anniversaries, self-indulgence, treat.<br><br>Country Manor: “Marketing objectives…to increase sales volume outside of a traditional corporate sales peak around the Christmas season”<br><br>Archers Schnapps: “[the advertising] aimed to take Archers beyond the cocktail image to a new drinking occasion’.<br><br>Fosters: “Comedy…presented a rare opportunity for sponsorship; few brands were associated with it, it gave permission for ‘ drinking occasion”<br><br>Archers: ““[the advertising] aimed to take Archers beyond the cocktail image to a new drinking occasion’<br><br>Lanson: One of the guidelines is to “Seek non-traditional situations with Champagne potential)” and “Groups trigger traditional associations; two people is enough”. (i.e., promote drinking Champagne in couples, not just in groups at celebrations). Campaign named ‘Why not?’ – i.e. ‘encapsulating the flexible attitude to champagne’. |
| Individual consumer-level | To prepare buyers before purchase | Stella Artois, 2002: “That promotions have worked so well is attributable in a large part to the role that advertising plays before consumers even step into the supermarket”<br><br>Glenmorangie: “We decided to launch the Christmas campaign in October for the first time, prior to the really busy Christmas onslaught, in order to prime the pump and build awareness”. |
| Individual consumer-level | Recruit new drinkers<br><br>Recruit younger drinkers | Miller lite: “If Miller Lite was to be a large profitable brand we had to attract these young heavy drinkers”<br><br>Glenmorangie: “Our primary target market was ABC1 males, 30-45 years old, but we were aiming more at the younger end of this group to encourage new drinkers into the market.”<br><br>Bowmore: ‘Rather than attempt to steal market share from the big boys, we wanted to find a way to introduce new drinkers to the market…”…”To sell, we had to make the product “accessible” to people who had previously overlook the malt whisky market” |

| | | |
|---|---|---|
| | | Budweiser: ‘If you want to recruit new drinkers then a strong on-trade presence I paramount’<br><br>Campari: “Encouragingly, the research also indicated that we were achieving the objective of attracting new, younger drinkers to the brand (Table 10.4).” (p. 122)<br><br>John Smiths: “In claimed behaviour, it seemed that the advertising had more effect on the younger drinker. The proportion of loyal drinkers who were aged 18-24 came back into line with other brands” (Pg. 22)<br><br>Castlemaine XXXX: “Growth comes from the steady flow of young drinkers with a strong preference for lager into the market. Drinkers tend to stick with lager as they grow older, and lager’s overall market share (currently about 40 per cent) appears to be moving inexorably towards its share of young drinkers; consumption (about 70 per cent for under 25s)”.<br><br>Tennents: “The objective for Tennents was to revitalise the brand amongst a younger audience, thus defending its position in spite of the dramatic changes affecting the market’”<br><br>Stella Artois: because of the strength of the brand, the promotions are leading to increased penetration, i.e. new buyers being attracted into the Stella franchise<br><br>Murphys: ‘Murphy’s has only attracted 1,000 regular Guinness drinkers. The remaining 238,000 new Murphy’s drinkers are most likely to have come from outside the stout category’. |
| **Brand/Product level** | Not sure what to call this –? | Johnnie Walker: “Johnnie Walker had to be not just a whisky brand but a global icon brand” [What this means is stated on p46 – e.g. like Coke, Nike etc – ‘brands that help [consumers] express who they want to be’]. |
| **Market** | Increase market share<br>Share of voice | Miller Lite: “Marketing objectives:…To grow Courage’s total draught standard lager share”<br><br>Boddington Bitter: “…has grown its share from 51% to 66% of all the case ales sold to …core accounts’. ‘The reasons to believe that the advertising is responsible for the growth in share that we have shown is that the growth occurred since the advertising was on air and no other factors can explain this growth’.<br><br>John Smith: “The 18-24s appear to be the most volatile group in terms of brand imagery and choice, and produced the greatest volume return for a given proportional change. If brand advertising could regain the |

| | | |
|---|---|---|
| | | loyalty of only a small proportion of these drinkers, share would regained, and with that would follow benefits for total pub revenue."<br><br>Paul Masson: "In assessing the brand and the contribution of advertising, it has always been recognised that sales and share have been the two key measures."<br><br>Budweiser: "We can demonstrate that the new advertising was driving these gains. First, there is a significant correlation between brand awareness and share of voice. Secondly the awareness shifts were greatest among our core target market of 18-24 year old adult lads".<br><br>Glenmorangie: '…we knew that the advertising would have to work extremely hard in order to win the aggressive share of voice battle waged every year in the whisky and spirits market at Christmas.'<br><br>Budweiser: Advertising reversed the decline in market share. 'As expected the first signs of recovery appeared in the on-trade'..It's interesting to note that these increases in market share lagged behind those in brand awareness that we observed earlier. Salience appeared to be driving market share, not vice versa' |
| | Develop new markets (similar to new customers) | Famous Grouse: 'The challenge was immense: to grow profits having exhausted our strategy of share gains through salesforce-led gains in distribution; to find a low-risk way of expanding into new markets [X167C] in the most cost-effective manner possible'.<br><br>Magners: "To deepen Magner's links with Rugby, it rebranded the Celtic Rugby Union League the Magners League, helping to extend the brand into Wales and lending even more gravitas plus the increased media exposure the coverage generated". |
| **Market** | To help market segmentation | "For the first time the stout market might begin to segment on a taste dimension for some consumers, and in doing so create space in people's minds for Murphy's". |
| **Market** | Sustain growth | Campari: "Our contention is that the advertising, firstly, by choosing the right medium for our message and, secondly, by creating a relevant, vivid and memorable campaign, has the most dramatic effect in the 1976 sakes off-take, and helped sustain growth thereafter"<br><br>Castlemaine: "While gratifying, advertising's greatest contribution is the strong base built for future profit – a secure and growing distribution base and brand franchise in a growing market." |

| | | |
|---|---|---|
| | | Johnnie Walker: “The brief from Diageo was twofold: to reverse the immediate fortunes of the brand in terms of sales; and to develop a future-proof, global communications strategy that would ensure sustained growth in all Johnnie Walker’s markets”<br><br>Budweiser: “Halt the drift of ‘adult lads’ from the brand, especially 18-24 year olds. These people are the heart of the on-trade. They also represent the future of the brand. If we can win them over when they reach adulthood then they’ll stick with us as they age.” |
| **Market** | Build future market | John Smiths: “The primary target market was defined as young bitter drinkers (18-30, C1C2D). They were the most frequent buyers, the future market, the least enthusiastic about John Smith’s Bitter.”<br><br>Paul Masson: “Furthermore, the credibility gained with the trade as a result of the success of Paul Masson California Carafe ensures a sympathetic response to Seagram wine products in the future”<br><br>Castlemaine: “Advertising has fuelled the rapid creation of strong consumer franchise and so a secure base for future profit in the growing lager market”. |
| **Market** | Develop new product category | Shakers cocktails: “Development of new brand AND product category – pre-mixed cocktail for take-home off-trade sales”.<br><br>Bacardi Breezer: “The strategic breakthrough was that there are occasions when drinkers, particularly, but not exclusively, in the on-trade, do not fancy a beer, they fancy a change, but cannot find anything suitable (because before Breezer there wasn’t anything). The vision was to reposition it as a ‘refreshing alternative’ alternative to beer.”<br><br>Magners: “Our intention was to build a new ‘’premium cider’ category that could command higher price points and drive value back into the category’. |
| **Market: Availability** | Increase distribution of product | Bud Ice: “Advertising strongly influenced both ROS and distribution of Bud Ice -and hence its market share. ‘An economic model shows that, at its peak, the advertising caused a 40% uplift in Bud Ice share”<br><br>Famous Grouse: “The efficiency with which we maintained our distribution is illustrated in a scatter plot, which shows the strong correlation between advertising investment and the ability of the brand to maintain distribution.” |

| | | |
|---|---|---|
| | | Stella Artois: "In the ontrade draught sector, Stella grows its market wherever it is present. This creates a more profitable product mix, benefiting both the brewer and the publican, and this in turn creates extra demand among the trade, thus increasing distribution" |
| | **Increase sales volume (volume growth and volume share)** | Volume explicitly mentioned in Campari, John Smiths, Shakers cocktails, Paul Masson; Country manor; Castlemaine XXXX; Lanson champagne; Croft Original sherry; Stella 1982; Boddingtons; John Smiths; Marstons Pedigree; Stella 1991; Murphys; Famous Grouse; Archers; Glenmorangie; Budweiser (2002); Magners cider; Jonnie Walker; Fosters; Glenmorangie 1576; Bowmore (SAW 2); Bowmore SAW 3; Grolsch (SAW3); Grolsch (SAW4)<br><br>Campari: "… an aging, middle class market, vulnerable to the effects of inflation and recession, did not provide sufficient scope for Campari's ambition for volume… That the effectiveness of Campari advertising can be measured in volume sales increases ahead of the total spirits market is impressive".<br><br>John Smiths Bitter: "The marketing objective was to increase volume sales" "…the 18-24s…???produced the greatest volume return for a given proportional change'.[check wording]<br><br>Shakers Cocktails:' Marketing objectives were to meet volume sales targets' [check wording]<br><br>Paul Masson: "Wine is increasingly an informal drink - both privately and socially. Importantly, however, new drinkers continue to enter the market every year. Volume growth therefore comes from increasing penetration as well as increasing frequency".<br><br>Country Manor: Marketing objectives: 1. To establish a new product which would enable Goldwell to increase sales volume outside of a traditional corporate sales peak around the Christmas season<br><br>Castlemaine XXXX: "The volume objectives required a brand with broad appeal among younger (18 to 30) drinkers on a national basis". "To make overall business sense, the new brand must produce substantial volume (and profit) over and above that which would result from the continued marketing of the existing lager portfolio"<br><br>Lanson "'Lanson has increased its prices faster than the Raft, and increased volume well ahead of the market."<br><br>Croft Original sherry: "'The combination of volume opportunity with improved margins means that a successful quality brand is a particularly profitable proposition' "Growth of the brand was also essential for Croft Original to exploit the seasonal Christmas volume opportunity" |

| | | |
|---|---|---|
| | | Stella 1982: ‘Volume sales increased 33% in 1982, and thereafter just kept on growing. By 1989 its volume sales were 406% up on the 1981 figure’. The volume growth outstripped the market, and Stella’s share of the market grew every year to 1987’.”<br><br>Boddington’s: “The task here was to grow volume outside Granada from a small base…” “‘Regression analysis of rate of sale from which the volume implication could be calculated’ – ‘Using this method we could see what rate of sale would have been if there had been no advertising for Canned Boddingtons’...’as we can see, volume as a result of advertising has been increasing’.”<br><br>John Smiths, 1993: “Marketing objectives: to achieve volume levels consonant with being a significant player in this market by Jan 1994”. Table 3 shows slight increase in on-trade volume sales (barrels), and share %.<br><br>Marstons Pedigree: “After the advertising started, volumes showed large and consistent increases compared with the equivalent non-advertised months”.<br><br>Stella Artois (1991): ‘Stella’s position in drinker repertoires was weakening as feared’…However more worrying was the impact on volume…between 1988 and 1990 Stella effectively ‘lost’ over 56,000 barrels’… “Volume increased from a low in 1991 of 46000 barrels, by 67% to a new record of 767000 barrels in 1995…” Check wording here:>Estimated that the advertising resulted in an uplift of nearly 280,000 barrels, around 20%.<br><br>Stella Artois (2000): “Stella was losing, not only share, but also volume” “£1m spent on advertising produces a direct volume uplift of 30,000 barrels, worth £12.6 million in increased revenue’.<br><br>Murphys: “Since the Murphy’s advertising campaign broke…the brand has achieved significant and consistent growth in both the on- and off=trade. In the on-trade Murphy’s volume has shown a significant increase from approximately 9000 barrels per month to approximately 14,000 barrels per month by the autumn of 1995. We can compare this volume growth to growth in the total market…Murphys volume share of stout has grown from around 11% to 13%. This is a very significant increase in the beer market’. “The advertising accounted for a 51% uplift in Murphy’s on trade volume sales and 97% off-trade”.<br><br>Famous Grouse: “The model estimates that Icon advertising generated 13% of volume sales in the long run”<br><br>Archers target audience: “A young stylish audience which gave the best volume and long-term opportunities’. |

| | | |
|---|---|---|
| | | Glenmorangie: “Our task was to increase volume sales…the results were impressive. In Year 1 volume sales…rose by 18.1% and value sales by 13%. This was significantly ahead of the market”’<br><br>Budweiser (2002): “We decided that …we needed to focus on the on-trade. This was for two reasons: volume of sales and because it is where brands are ‘adopted’”.<br><br>Famous Grouse (1995-7): , “Whisky brands are very reliant on a small number of heavy, and increasingly ageing, consumers, to provide the majority of volume. The Famous Grouse was no exception…Effects of campaign: “Our 55+ drinkers, disproportionately important in volume terms increased by 144,000 between 1997 and 1997” (competitors lost drinkers)”.<br><br>Magners cider: “We knew there would be a significant volume opportunity if we could persuade drinkers in the UK to introduce or for lapsed drinkers reintroduce, cider into their drinking repertoire. The volume opportunity existed across all age groups, so we intentionally did not focus Magners towards a youth audience where lager brands focus their attention’.”<br><br>Glenmorangie 1576: “We focused our communication on a more mainstream drinker (for volume purposes) ) whilst not alienating the Connoisseur”. “There was an increase in volume share, and brand share by volume” (Figs 3-5).<br><br>Jonnie Walker whisky: “…Johnnie Walker was on red alert. In the three years since 1996, volume sales had dropped 14%. The brand was also steadily losing market share…”. Effects of advertising campaign include: “Increase in volume sales of 48% (p65); Increase in volume share of 3.9% (p65).” ““Johnnie Walker volume increased 3% on strong advertising and promotion in a declining whisky category”“<br><br>Fosters lager: “Over six years from 2004 to 2010, lager volume dropped by 19%, which meant Britons were drinking 1.3 billion fewer pints a year – the equivalent of wiping out the top two lager brands combined’. “Business objectives: 1. Make Foster’s the number one volume brand of standard lager in the off-trade by 2012… heading into the summer, with a football World Cup only 20 weeks away, a massive volume opportunity beckoned…‘…Foster’s grew from third place in the off-trade, to an outright number one in both volume and value…This was achieved without extra discounting. In fact average price paid increased 14%. “<br><br>Bowmore (SAW 2): “Bowmore wanted to build its volume and market share – but not at the expense of value”. “Following the launch of the campaign, sales growth was immediate…fastest growing malt for the year. Dramatic increases were made in terms of both volume and value…the effect of brand on the increasing value of Bowmore sales was even more dramatic…Bowmore succeeded in increasing its market share.” |

| | | |
|---|---|---|
| | | Bowmore (SAW 3): "…the brand achieved…growth in volume sales of 5.3%..other data on sales are presented volume increases since start of campaign.<br><br>Scottish leader: "We contend that the effect of the advertising was both to increase sales in existing outlets and help secure additional distribution, generating further sales…one way to…isolate advertising's effect is to compare volumes between areas with and without advertising."<br><br>Grolsch (SAW 3): Advertising objectives include "Double sales to reach half a million barrels by 2002…Grolsch's main target was to sell 500 thousand barrels by 2002. It actually exceeded this by 75,000'.<br><br>Grolsch (SAW4): "Target 3: Double volumes by 2006" |
| | **Increase rate of Sale/purchase** | Archers schnapps: 'Rate of sale in the advertised periods is consistently higher than in the surrounding months…High correlations between TV exposure and rate of sale (p419). "…direct connection between the advertising and the sales uplifts'<br><br>Grolsch: 'The most concrete measure of increased consumer demand is rate of purchase. Grolsch's rate has more than trebled since campaign launch…Put simply, consumers are demanding the brand more than ever'. |
| | **Extend drinker's drinking repertoires** | Magners: "We knew there would be a significant volume opportunity if we could persuade drinkers in the UK to introduce or for lapsed drinkers reintroduce, cider into their drinking repertoire."<br>Stella Artois: Motivation for campaign: 'Stella's position in drinker repertoires was weakening as feared' |
| **Brand/Product level** | **Build/strengthen brand equity** | Scottish Leader Whisky: "Advertising played a key part in building that brand equity'<br>"The Famous Grouse …campaign was launched…to build brand equity"<br><br>Glenmorangie: "We found that Glenmorangie's brand equity had unquestionably increased over the advertising period…we concluded that the brand equity rises must be due to the advertising'.<br>Fosters: "Comedy…presented a rare opportunity for sponsorship; few brands were associated with it, it gave permission for ' drinking occasion' and it was an equity that the brand had strongly delivered against during its heyday…"<br><br>Budweiser: It also appears that there was a halo effect because product measures lifted as well ('particularly refreshing' and 'good quality') even though we didn't talk about them in our ads. Once we had got people to like our ads and our brand again, they started to think positively about it in every sense'. |

| | | |
|---|---|---|
| | | Marstons Pedigree: “Halo” effect reported in the case of Marstons Pedigree also. |
| **Brand/Product level** | **Create/strengthen brand and/or product identity/personality/image** | Grolsch: ‘Dutchness’; ‘laidback, easygoing approach to life’. ‘This very appealing character trait would form the basis of an appealing brand personality.<br>Croft original Sherry: “To continue to present paleness as a positive virtue”<br>Hofmeister: “Given the need to create a new, confident image for the brand, TV provided the most intrusive and influential way to reach our young drinkers.”<br>Castlemaine: *‘The image which the campaign has created is of an authentic Australian lager, drunk by genuine Australians who know a good lager… Castlemaine is seen as a true export of Australia, despite knowledge that the product is UK brewed.*<br>Stella Artois: “For Stella’s advertising to work …It had to elevate Stella’s image for strength above Holsten Pils, and maintain a lead over the ‘macho’ positioned Tennents Extra. This would secure the brand’s appeal to ‘Headbangers’”.<br>Famous Grouse: “The Icon campaign was successful at building ad awareness and brand image” |
| | **Communicate or imply strength of product** | Stella Artois: “If the campaign was working correctly, it should have been building a strong image for Stella on the areas of flavour, strength and fashion appeal, as well as a reputation for being more expensive…some of the pertinent quotes were ‘It’s recognised as being very strong, and it is…. It’s one of the strongest of all’.<br>…It moved from behind Holsten Pils (-8% in terms of perceived strength to ahead by +9%)”.<br><br>Stella Artois: “For Stella’s advertising to work …It had to elevate Stella’s image for strength above Holsten Pils, and maintain a lead over the ‘macho’ positioned Tennents Extra. This would secure the brand’s appeal to ‘Headbangers’”.<br><br>Miller Lite: The words ‘from America’ were subsequently dropped from the campaign, as it undermined the need to communicate the strength of the lager. It had a ‘damaging effect on the brand’ s perceived alcoholic strength’. |
| **Brand/Product level** | **Reposition product** | Bacardi Breezer: “The vision was to reposition it as a ‘refreshing alternative’  alternative to beer.’ |
| | **To support other promotions** | Stella Artois, 2002: “That promotions have worked so well is attributable in a large part to the role that advertising plays before consumers even step into the supermarket” |

| | | |
|---|---|---|
| **Brand/Product level** | **Use advertising to maintain or increase higher price/brand value**<br><br>**Premiumisation** | Famous Grouse: Advertising objectives include: "Build a price premium against competitors in the off trade"…"The price premium versus Teachers increased post-advertising…Commanding a price premium is even more important for profitability in the spirits category, compared to other food and drink categories, because of the relatively high excise duty fixed cost."<br><br>Stella Artois: "the premium price ideally pays for the advertising which tells the consumer the product costs more in the first place"<br><br>Archers Schnapps: "on-trade pricing rose in an almost linear manner…4% above the underlying price increases in the spirits market as a whole…change in price acts as another measure of advertising's contribution to the value of the brand.<br><br>Fosters: Strong brands are less price sensitive, and require less discounting in order to drive sales volume.(p15). The most important ingredient in this process is emotional bonding. If people really like a brand, they're willing to pay more for it.'..<br><br>Archers: "…allowing us to continue to charging a full-spirit premium on a half-strength measure'."<br><br>Archers: "on-trade pricing rose in an almost linear manner…4% above the underlying price increases in the spirits market as a whole…change in price acts as another measure of advertising's contribution to the value of the brand.'<br><br>Johnnie Walker: Measures of effectiveness include "Brand stretch into more premium segments" .<br><br>Tennents: '…the [Red T] symbol's power is itself a direct result of advertising…we are entitled to regard these other factors as manifold effects of the Red T campaign: The launch of the new can…The launch of T in the Park …a platform for premiumisation. 'The long-term success of Tennents Lager branding now offers a way to extend the brand's provenance into the premium category. |
| | **Create property for future use** | John Smith Bitter: Advertising added the word 'widget' to popular culture – a 'property for JS that can be exploited in the future'.<br><br>Fosters: '…Good Call' [campaign] paved the way for a successful launch of [Foster's] Gold.' |

| | | |
|---|---|---|
| **Brand/Product level** | **Protect brand ; prevent decline in brand** | Hofmeister: "Non-drinkers of Hofmeister… …were now reluctant to criticise the brand." (p81)<br><br>Murphys: The brand was in decline, facing potential delisting. 'Whitbread's salesforce was finding it difficult to drive distribution…we suspected that, if this decline was allowed to contribute, the brand's fortunes would begin to spiral down'.<br>Famous Grouse "…A 14% decline in sales would have occurred in the on trade 1997-2005. This is equivalent to £189m of retail sales"<br><br>Budweiser: "The econometric model for this sector shows that without the advertising, Budweiser's share would actually have suffered a significant decline'."<br><br>Tennents: 'The objective for Tennents was to revitalise the brand amongst a younger audience thus defending its position in spite of the dramatic changes affecting the market'.<br><br>Tennents: "It is less straightforward to demonstrate the effects of advertising for brands that occupy market position, yet such cases probably account for most of the advertising we see. This 'defensive role' for advertising is hugely important in sustaining relevance for established brands but is harder to isolate, since established brands, by their nature, are more resistant to short-term fluctuations in awareness, image and share" |
| **Brand/Product level** | **Increase sales of non-advertised products; spillover to category** | Boddington: The advertising also increased the sales of the 'old' can, which was not advertised – 'This suggests that there is a common cause for both these growth rates…we believe the common cause is the advertising'.[p150]<br><br>Stella Artois: '…the brand is so strong that it premiumises the product mix: where Stella is present the ratio of premium lager sold to standard is higher than where it is not"<br><br>Bud Ice: The advertising also benefitted Budweiser – the parent. It shifted both spontaneous awareness and sales…at one point Bud Ice ads boosted Bud's share by 14%") "As importantly, we have shown that Bud Ice advertising has increased sales of the parent brand'"<br><br>Bowmore: "we decided to focus our efforts on Scotland and, in particular, at driving off-trade volume. The theory being that high value expressions would benefit obliquely too" |
| **Company** | **Improve value of parent company** | Scottish Leader whisky: 'The advertising…acted as a rallying point for employees. It sent out a signal to the trade. It contributed to Burn Stewart's takeover in January 2003 by Angostura.' |

| | | |
|---|---|---|
| | | Boddingtons Bitter: 'Whitbread has benefitted corporately from the success of Boddingtons and its advertising: 'As a result our marketing pedigree has been notched up which is of value to the whole Plc'". <br><br> Johnnie Walker Whisky: "An additional success of Keep Walking was to raise Johnnie Walker's profile with Diageo's financial stakeholders' <br><br> Marstons Pedigree: 'As a publicly quoted company, Marston's last financial year was its most profitable ever and the informed view was that the company's advertising was a significant factor'. Spillover effect of the advertising to consumer perceptions of Marston's pubs also noted. |
| **Business/community** | **Employment** | Bacardi Breezer: "'Moreover the value of the investment and new jobs is multiplied two to three times when one looks at the economic impact on the local community' <br><br> 'In the mid 1970s, two unbelievably hot summers, rampant inflation and rising unemployment heralded the arrival of a new breed of premium lager drinker. These young men sought nirvana through bottles of Holsten Pils, or cans of Fosters, and their habit of falling over at the end of each session earned them the title 'Headbangers'….Then in 1980 Bass launched Tennents Extra. It was nationally availably, chilled on draught right from the start, had unequivocally macho advertising, and was not that expensive. Pretty soon 'heads' were being 'banged' all over the UK'. |
| **Business/community** | **Block competition** | Archers: "Advertising increases the likelihood of Archers being asked for by name, which decreases the risk of delisting, and protects it against competitive launches. (p421). 'This secondary effect of advertising is a strong reason why there have been no notably successful schnapps launches during Archers lifetime'. |
